# The model for predicting the central lymph node metastasis in cN0 papillary thyroid microcarcinoma with Hashimoto’s thyroiditis

**DOI:** 10.3389/fendo.2024.1330896

**Published:** 2024-04-30

**Authors:** Yuyang Lin, Na Cui, Fei Li, Yixuan Wang, Bei Wang

**Affiliations:** ^1^ Department of Ultrasound, The First Affiliated Hospital of Shandong First Medical University and Shandong Provincial Qianfoshan Hospital, Jinan, Shandong, China; ^2^ Department of Medical Ultrasound, The 960th Hospital of the Chinese People's Liberation Army Joint Logistic Support Force, Jinan, Shandong, China

**Keywords:** papillary thyroid microcarcinoma, central lymph node metastasis, Hashimoto's thyroiditis, prophylactic central lymph node dissection, nomogram

## Abstract

**Background:**

The relationship between Hashimoto’s thyroiditis (HT) and papillary thyroid microcarcinoma (PTMC) is controversial. These include central lymph node metastasis (CLNM), which affects the prognosis of PTMC patients. This study aimed to establish a predictive model combining ultrasonography and clinicopathological features to accurately evaluate latent CLNM in PTMC patients with HT at the clinical lymph node-negative (cN0) stage.

**Methods:**

In this study, 1102 PTMC patients who received thyroidectomy and central cervical lymph node dissection (CLND) from the First Affiliated Hospital of Shandong First Medical University from January 2021 to December 2022 and the 960th Hospital of PLA from January 2021 to December 2022 were jointly collected. The clinical differences between PTMCs with HT and those without HT were compared. A total of 373 PTMCs with HT in cN0 were randomly divided into a training cohort and a validation cohort. By analyzing and screening the risk factors of CLNM, a nomogram model was established and verified. The predictive performance was measured by the receiver operating characteristic (ROC) curve, calibration curve, and clinical decision curve analysis (DCA).

**Results:**

The ratio of central lymph node metastasis (CLNMR) in PTMCs with HT was 0.0% (0.0%, 15.0%) and 7.7% (0.0%, 40.0%) in the non-HT group (*P*<0.001). Multivariate logistic regression analysis showed that age, gender, calcification, adjacent to trachea or capsule, and TPOAB were predictors of CLNM in PTMCs with HT. The areas under the curve (AUC) of the prediction models in the training cohort and the validation cohort were 0.835 and 0.825, respectively, which showed good differentiation ability. DCA indicates that the prediction model also has high net benefit and clinical practical value.

**Conclusion:**

This study found that CLN involvement was significantly reduced in PTMC patients with HT, suggesting that different methods should be used to predict CLNM in PTMC patients with HT and without HT, to more accurately assist preoperative clinical evaluation. The actual CLNM situation of PTMCs with HT in cN0 can be accurately predicted by the combination of ultrasonography and clinicopathological features.

## Introduction

The incidence of papillary thyroid carcinoma (PTC) has significantly increased due to the widespread use of high-frequency ultrasound (1), leading to an increased detection rate of papillary thyroid microcarcinoma (PTMC) patients with a diameter ≤1cm. According to the Global Cancer Report published by WHO in 2014, more than 50% of new cases of thyroid cancer are accounted for by PTMCs (2, 3). However, current studies have shown that PTMCs are less aggressive and have a 10-year survival rate of over 91% (3, 4).

Takamura Y et al. found that the incidence of central lymph node metastasis (CLNM) in PTMC patients was as high as 30%-64% (5). CLNM of PTMC usually occurs initially in the central compartment and is an important prognostic factor associated with tumor recurrence. The 2015 American Thyroid Association (ATA) guidelines recommend therapeutic central lymph node dissection (CLND) in patients with lymph node metastasis (LNM) (6), while prophylactic central lymph node remains controversial for patients with cN0. Several studies have reported significant improvements in 10-year disease survival and local area control in PTC with bilateral pCND followed by personalized radioiodine therapy (7). Furthermore, studies have indicated that performing pCND in stage clinical lymph node-negative (cN0) patients does not significantly reduce the risk of local recurrence. However, pCND is an invasive and traumatic procedure that can lead to serious complications such as permanent hypoparathyroidism and recurrent laryngeal nerve damage (6). Nevertheless, approximately 53.5% of cN0 patients exhibit latent CLNM (8), which may impact TNM staging and treatment decisions for certain patients (9). Therefore, accurate preoperative diagnosis of CLNM is crucial.

Conventional ultrasound has limitations in evaluating central lymph nodes (CLNs) due to the structural characteristics of the neck and acoustic principles, resulting in a sensitivity below 50% (10). Consequently, accurately assessing CLNM becomes challenging. To address this issue, stratified management based on ultrasound features combined with prognostic factors associated with increased risk of metastasis and recurrence (e.g., age, gender, size, extrathyroidal invasion, evidence of invasive cytology) should be considered for PTMCs in cN0 patients. Those at high risk for CLNM are more likely to undergo thyroidectomy and pCND procedures.

Hashimoto thyroiditis (HT), HT is known as chronic lymphocytic thyroiditis and often coexists with PTC/PTMC (11). Some studies have suggested that PTMC patients with HT have lower clinical invasiveness and better prognosis, and are negatively correlated with lymph node metastasis (12). However, its association with PTMC remains unproven. Exploring clinical differences between PTMCs with HT versus those without HT could provide insights into lymph node involvement among these two groups. If there are distinct variations in CLNM presentation between these groups then different models can be adopted to predict the risk of CLNM for PTMCs accordingly.

The objective of this study was to establish an implicit CLNM prediction model based on independent risk factors extracted from cN0 PTMC patients with HT, which can help surgeons develop individualized treatment plans for such patients.

## Materials and methods

### Patients

A total of 1250 patients with PTMC pathologically diagnosed in the First Affiliated Hospital of Shandong First Medical University from January 2021 to December 2022 and 960th Hospital of PLA from January 2022 to December 2022 were jointly collected and retrospectively analyzed.

**Figure 1:**
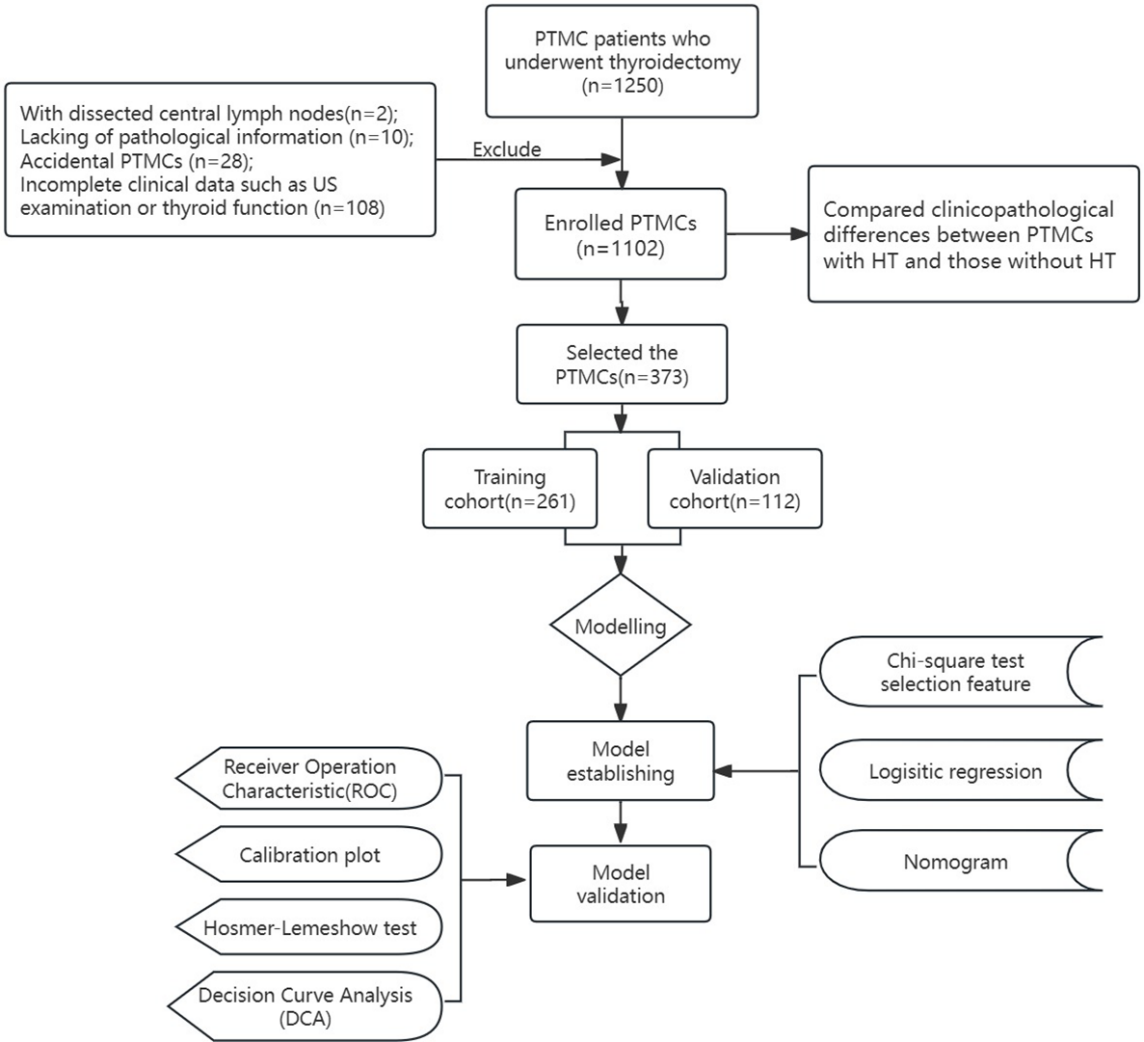
Flow diagram of the study design.

The inclusion criteria were: (1) age >18 years old; (2) performing thyroidectomy and CLND; (3) PTC confirmed by histopathology. The exclusion criteria are (1) previous history of thyroid surgery; (2) combined with other malignant diseases; (3) Pathological findings showed thyroid carcinoma (TC) with other pathologic types except PTC, such as follicular or anaplastic thyroid carcinoma; (4) incomplete clinical data such as preoperative laboratory tests or ultrasound; (5) Preoperative clinical findings of lymph node metastasis or distant metastasis; (6) patients without CLND; (7) received TSH or other drug suppression therapy before surgery. (**Figure 1**. Flow diagram of the study design).

After excluding 148 patients who did not meet the criteria, 1102 PTMC patients were included. A total of 373 PTMC patients with HT were included in the study, they were randomly divided into the training cohort (261 patients) and the validation cohort (112 patients) at a ratio of 7:3.

### Variable definition and evaluation

The clinicopathological features of the patients were analyzed. They included gender, age, ultrasound features (size, echogenicity, calcification, margin, shape, anteroposterior and transverse diameter ratio (AT), tumor location, presence or absence of HT ultrasound features), and thyroid function tests including free triiodothyronine (FT3), free thyroxine (FT4), thyroid stimulating hormone (TSH), thyroglobulin antibodies (TGAB) and thyroid peroxidase antibodies (TPOAB). And pathological characteristics (multifocal or unifocal, unilateral or bilateral, extrathyroidal invasion, BRAF^V600E^ gene mutation, pathological type, bilateral CLNM, and pathological diagnosis of HT). The number of surgically dissected CLNs, the number of CLNM, and the CLNMR (the number of metastatic lymph nodes per patient divided by the number of CLNS removed) were recorded. The levels of TSH, TPOAB, and TGAB were grouped according to previous studies (11).

The thyroid surgery in this study followed the 2016 Chinese Expert Consensus on the Diagnosis and Treatment of Thyroid Micropapillary Carcinoma. The total thyroidectomy or thyroid lobectomy was performed for the primary tumors, and the dissection of the secondary lesions (lymph nodes) followed the principle of individualized treatment. It is generally recommended to perform the CLND, lateral cervical lymph node dissection was performed only when there was evidence of lateral cervical lymph node metastasis before or during operation.

Tumor size is the maximum tumor diameter measured by preoperative ultrasound images. Ultrasound features: lesion echogenicity is divided into two subgroups: hyperechoic/isoechoic and hypoechoic/very hypoechoic; calcification (no calcification, macrocalcification, and microcalcification); shape (regular/irregular); margin (well-defined/ill-defined). Lesions with A/T>1 showed that the anteroposterior is greater than the transverse diameter in two-dimensional ultrasound images. The tumors are classified according to the location in the upper, middle, and inferior, isthmus, and whether the tumor is adjacent to the trachea or the capsule (the tumor edge is less than 2mm from the trachea or capsule). The above ultrasonic features were determined by two physicians with more than ten years of working experience in the ultrasound department of the hospital. If the results are inconsistent, the senior physician rules.

More than one PTC lesion in the thyroid gland is defined as multifocal. The presence of more than one PTC lesion in one gland at the same time is considered to be unilateral multifocal, and the presence of more than one PTC lesion in the bilateral lobe or unilateral lobe plus isthmus is considered to be bilateral multifocal. In the presence of multifocal lesions, the size and location of the tumor are determined by the diameter and location of the largest dominant lesion. When the main lesion occupies 2 adjacent parts, the location of the tumor is determined by the part containing more than two-thirds of the tumor volume.

We adopted the overall definition of chronic lymphocytic thyroiditis (CLT), including (I) Elevated levels of thyroid peroxidase antibodies. (II) Diffuse heterogeneity on ultrasonography. (III) Histopathological diagnosis of diffuse lymphocytic thyroiditis. To avoid selection bias, HT was diagnosed when any of the above conditions were met in this study. The pathological types were divided into low-risk subgroup (classic, follicular subtype, encapsulation, clear-cell, eosinophilic cell, Warthin neoplastic cell) and high-risk subgroup (diffuse sclerosis type, solid growth, tall cell, columnar and Hobnail cell).

### Statistical analysis

SPSS26.0 and R Studio (R.4.2.2) were used to analyze the data. When *P*<0.05, the analysis was considered statistically significant. A chi-square test was conducted for categorical variables, and an independent t-test or Mann-Whitney U test was conducted for continuous variables.

The chi-square test was used to select the characteristics of the training cohort, and then the independent risk factors for CLNM were obtained by regression analysis. A nomogram model for predicting CLNM was established, and the calibration curves were analyzed. In addition, the receiver operating characteristic (ROC) curve, C index, and Hosmer-Lemeshow good of fit test were used to evaluate the value of the predictive model, and decision curve analysis (DCA) calculated the clinical net benefit of the predictive model.

## Results

### Basic clinical features of HT group and non-HT group

PTMC patients were stratified based on their HT status and compared between groups (**Table 1**). The results revealed statistically significant differences in gender, A/T, BRAF^V600E^ gene mutation, number of dissected lymph nodes, and number of CLNM and CLNMR between the two groups (all *P*<0.05). Specifically, the non-HT group had a significantly lower number of dissected lymph nodes compared to the HT group (*P*<0.001), while the non-HT group exhibited a markedly higher CLNMR than the HT group (*P*<0.001).

**Table 1:** Clinicopathological features of PTMC patients with HT or without HT.

Variables	Without HT (n=729)	With HT (n=373)	*P*
Age (y) <45/≥45	325/404	163/210	0.780
**Gender** M/F	235/494	46/327	<0.001
Ultrasonic Characteristics			
** Echo** (hyperechoic or isoechoic/ very hypoechoic or hypoechoic)	61/668	29/344	0.734
** Calcification** (none/macro-/ micro-)	314/118/297	140/64/169	0.202
** Margin** (well-defined/ill-defined)	107/622	47/326	0.347
** Shape** (regular/irregular)	95/634	46/327	0.742
** A/T** (≤1/>1)	222/507	139/234	0.023
** HT of Ultrasound** (no/yes)	695/34	222/151	<0.001
Location			
(upper/middle/ inferior/isthmus)	171/313/159/86	76/158/90/49	0.570
(adjacent to trachea or capsule/none)	251/478	124/249	0.712
Pathology			
** Multifocality** (no/yes)	453/276	243/130	0.327
** Bilateral** (no/yes)	570/159	288/85	0.712
** Extrathyroidal invasion** (No/Capsule Contact/ETE)	221/452/56	122/222/29	0.703
** BRAF^V600E^ mutation** (no/yes)	53/676	42/331	0.026
**High-risk pathological type** (no/yes)	724/5	366/7	0.071
** CLN examined**	5 (3-9)	9 (5-15)	<0.001
** CLNMs**	0 (0-2)	0 (0-1)	0.002
** CLNMR**	7.7% (0.0%-40.0%)	0.0% (0.0%-15.0%)	<0.001

Tumor size, CLN examined, and CLN positive are all expressed as the median (Q1-Q3).

### Baseline clinical characteristics of PTMCs with HT

The clinical characteristics of 373 PTMCs with HT in cN0 were analyzed. Among them, 139 patients presented with CLNM while 234 patients did not. All patients were randomly divided into a training cohort (261 cases) and a validation cohort (112 cases) at a ratio of 7:3 for comparison of their clinical characteristics. **Table 2** demonstrates that there were no significant differences in baseline characteristics between the training and validation cohorts (all *P*>0.05), and the similarity of clinical information proves their rationality as training and validation cohorts.

**Table 2:** Clinical baseline analysis of cN0 PTMCs with HT in the training and validation cohorts.

Variables	Subgroup	No. of patients(PTMC with HT)		*P*
		Training Cohort (n=261)	Validation Cohort (n=112)	
**Age (year)**		47	48	0.618
	<45	117	46	0.503
	≥45	144	66	
**Gender**	male	30	16	0.452
	female	231	96	
**Race**	Chinese	261	112	~
**Tumor size (mm)**		6 (5-8)	6 (5-8)	0.525
**Multifocality**	no	165	78	0.233
	yes	96	34	
**Extrathyroidal invasion**	no	87	35	0.614
	capsule contact	156	66	
	ETE	18	11	
**Location**	upper	55	21	0.724
	middle	112	46	
	inferior	63	27	
	isthmus	31	18	
**Echogenicity**	hyperechoic or isoechoic	20	11	0.618
	very hypoechoic or hypoechoic	241	101	
**Calcification**	none	97	43	0.922
	macro-	44	20	
	micro-	120	49	
**Margin**	well-defined	30	17	0.326
	ill-defined	231	95	
**Shape**	regular	29	17	0.273
	irregular	232	95	
**A/T**	≤1	93	46	0.319
	>1	168	66	
**BRAF^V600E^ mutation**	no	29	13	0.890
	yes	232	99	
**High-Risk Pathological type**	no	256	110	0.932
	yes	5	2	
**TSH (μIU/mL)**	≤0.35	7	3	0.999
	0.35-5	238	102	
	>5	16	7	
**FT3 (pmol/L)**		4.46	4.39	0.842
**FT4 (pmol/L)**		17.16	16.92	0.810
**TGAB (IU/mL)**	normal	116	53	0.517
	115-575	92	39	
	575-1150	31	8	
	≥1150	22	12	
**TPOAB (IU/mL)**	normal	127	56	0.925
	34-170	71	30	
	170-340	50	19	
	≥340	13	7	
**CLN examined**		9 (5-15)	9 (5-14)	0.907
**CLN positive**		0 (0-1)	0 (0-2)	0.750

A/T, aspect ratio; ETE, extrathyroidal extension.

Tumor size, CLN examined, and CLN positive are all expressed as the median (Q1-Q3). Age, FT3 and FT4 are all expressed as the median.

### Screening of risk features affecting CLNM in patients with HT PTMC

The training cohort was divided into a CLNM group (100 cases) and a non-CLNM group (161 cases) to compare variables between these two groups and identify characteristic factors influencing CLNM in PTMCs with HT. As shown in **Table 3**, age, gender, calcification, multifocality, bilateral lobes, size, and adjacent to trachea or capsule all demonstrated significant correlations with CLNM development in PTMCs with HT(*P*<0.05).

**Table 3:** Screening of risk features affecting CLNM in cN0 PTMCs with HT in the training cohort.

Variables	Subgroup	No. of patients(PTMC with HT)		*P*
		Without CLNM(n=161)	With CLNM(n=100)	
**Age (year)**	<45	62	55	**0.009**
	≥45	99	45	
**Gender**	male	12	18	**0.009**
	female	149	82	
**Tumor size (mm)**		6 (4-8)	7 (5-8)	**0.001**
**Multifocality**	no	110	55	**0.030**
	yes	51	45	
**Bilateral**	no	131	70	**0.034**
	yes	30	30	
**Extrathyroidal invasion**	no	60	27	0.176
	capsule contact	92	64	
	ETE	9	9	
**Adjacent trachea or capsule**	no	71	14	**<0.001**
	yes	90	86	
**Echogenicity**	hyperechoic or isoechoic	11	4	0.618
	very hypoechoic or hypoechoic	150	96	0.339
**Calcification**	none	81	16	**<0.001**
	macro-	36	8	
	micro-	44	76	
**A/T**	≤1	60	33	0.484
	>1	101	67	
**BRAF^V600E^ mutation**	no	21	8	0.208
	yes	140	92	
**High-Risk Pathological type**	no	158	98	0.938
	yes	3	2	
**TSH (μIU/mL)**	≤0.35	5	2	0.862
	0.35-5	146	92	
	>5	10	6	
**FT3 (pmol/L)**		4.83	4.81	0.556
**FT4 (pmol/L)**		17.16	16.87	0.134
**TGAB (IU/mL)**	normal	73	43	0.968
	115-575	55	37	
	575-1150	19	12	
	≥1150	14	8	
**TPOAB (IU/mL)**	normal	71	56	0.240
	34-170	46	25	
	170-340	34	16	
	≥340	10	3	

A/T, aspect ratio; ETE, extrathyroidal extension.

Tumor size, CLN examined, FT3, and FT4 are all expressed as the median. The bold values indicate statistical significance.

### Univariate and multivariate analyses of CLNM in cN0 PTMC with HT

**Figure 2:**
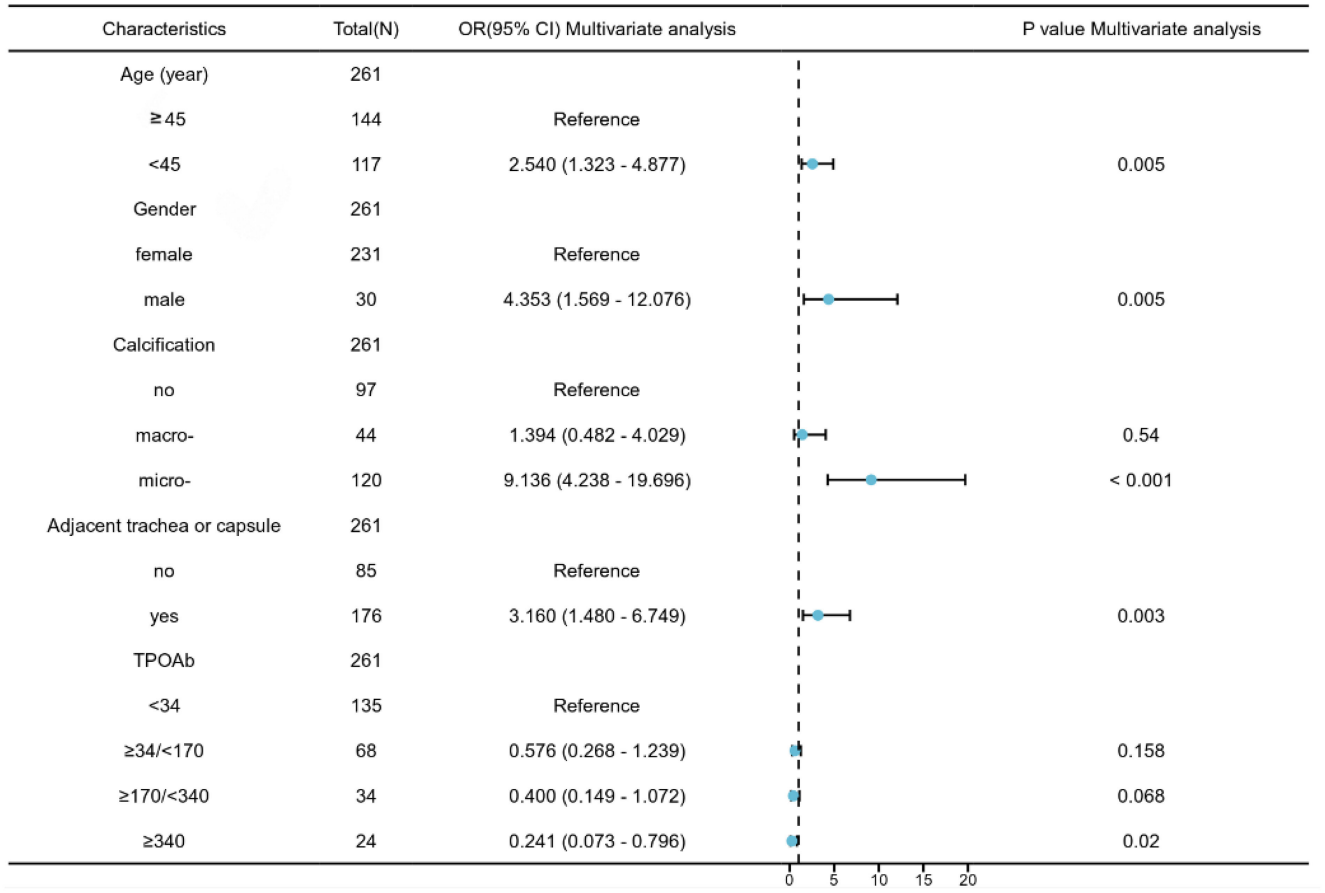
The forest plots for predicting CLNM in PTMCs with HT based on multivariate analysis.

The independent predictors of CLNM in PTMCs with HT in the training cohort were analyzed by logistic regression. As shown in **Table 4**, the results of univariate logistic regression analysis show that the factors are consistent with **Table 3**. Age, gender, calcification, multifocality, bilateral lobes, size, and whether the tumor is adjacent to the trachea or capsule were all predictors of CLNM(*P*<0.05). TPOAB (*P*<0.1) was also included in the multivariate logistic regression analysis. Further multivariate logistic regression analysis finally showed that five features are significantly correlated with CLNM in PTMC patients with HT, age <45 years (*B*=0.842, OR:2.54, *P*=0.005), male (*B*=1.314, 0R:4.35, *P*=0.005), microcalcification (*B*=2.346, OR:9.14, *P*<0.001) and adjacent to trachea or capsule (*B*=1.234, OR:3.116, *P*=0.003) are predictive risk factors for CLNM in PTMCs with HT, while TPOAB levels higher than 340 IU/mL (*B*=-1.314, OR: 0.2419.14, *P*=0.02) were protective factor for CLNM. With the increase in TPOAB level, the incidence of CLNM decreased (**Figure 2**).

**Table 4:** Univariate and multivariate analysis of PTMCs with HT in cN0 for CLNM.

Variables	Total(N)	Univariate analysis		Multivariate analysis		
		Odds Ratio (95% CI)	*P*	Odds Ratio (95% CI)	*B*	*P*
**Age (year)**	261					
≥45	144	Reference		Reference		
<45	117	1.952 (1.177 - 3.237)	**0.010**	2.540 (1.323 - 4.877)	0.842	**0.005**
**Gender**	261					
female	231	Reference		Reference		
male	30	2.726 (1.251 - 5.937)	**0.012**	4.353 (1.569 - 12.076)	1.314	**0.005**
**Calcification**	261					
no	97	Reference		Reference		
macro-	44	1.125 (0.442 - 2.866)	0.805	1.394 (0.482 - 4.029)	0.516	0.540
micro-	120	8.744 (4.555 - 16.787)	**< 0.001**	9.136 (4.238 - 19.696)	2.346	**< 0.001**
**Echogenicity**	261					
hyperechoic or isoechoic	15	Reference		-		-
very hypoechoic or hypoechoic	246	1.760 (0.545 - 5.686)	0.345	-		-
**A/T**	261					
>1	168	Reference		-		-
≤1	93	0.829 (0.490 - 1.402)	0.484	-		-
**Adjacent trachea or capsule**	261					
no	85	Reference		Reference		
yes	176	4.846 (2.543 - 9.235)	**< 0.001**	3.160 (1.480 - 6.749)	1.234	**0.003**
**Multifocality**	261					
no	165	Reference		Reference		
yes	96	1.765 (1.054 - 2.954)	**0.031**	1.841 (0.729 - 4.650)		0.197
**Bilateral**	261					
no	201	Reference		Reference		
yes	60	1.871 (1.044 - 3.354)	**0.035**	0.895 (0.310 - 2.585)		0.838
**Extrathyroidal invasion**	261					
no extrathyroidal invasion	87	Reference		Reference		
only capsule contact	156	1.546 (0.887 - 2.693)	0.124	1.050 (0.495 - 2.226)		0.898
ETE	18	2.222 (0.794 - 6.222)	0.128	1.209 (0.308 - 4.741)		0.786
**BRAF^V600E^ mutation**	261					
no	29	Reference				
yes	232	1.725 (0.733 - 4.059)	0.212	-		-
**Tumor Size**	261	1.228 (1.082 - 1.393)	**0.001**	1.084 (0.912 - 1.288)		0.360
**High-Risk Pathological type**	261					
no	256	Reference				
yes	5	1.075 (0.176 - 6.547)	0.938	-		-
**T3**	261	1.094 (0.888 - 1.347)	0.399	-		-
**T4**	261	1.047 (0.972 - 1.126)	0.224	-		-
**TSH**	261					
≤0.35	4	Reference				
0.35-5	241	1.885 (0.193 - 18.393)	0.585	-		-
>5	16	1.800 (0.151 - 21.477)	0.642	-		-
**TPOAb**	261					
<34	135	Reference		Reference		
34-170	68	0.658 (0.359 - 1.208)	0.177	0.576 (0.268 - 1.239)	-0.321	0.158
170-340	34	0.703 (0.322 - 1.535)	0.376	0.400 (0.149 - 1.072)	-0.907	0.068
≥340	24	0.429 (0.160 - 1.149)	0.092	0.241 (0.073 - 0.796)	-1.356	**0.020**
**TGAB**	261					
<115	139	Reference				
115-575	94	1.361 (0.795 - 2.327)	0.261	-		-
575-1150	18	1.169 (0.426 - 3.208)	0.762	-		-
≥1150	10	1.224 (0.330 - 4.548)	0.762	-		-

“-” representing meaninglessness. The bold values indicate statistical significance.

### Construction and validation of nomogram for predicting CLNM in PTMC with HT

**Figure 3:**
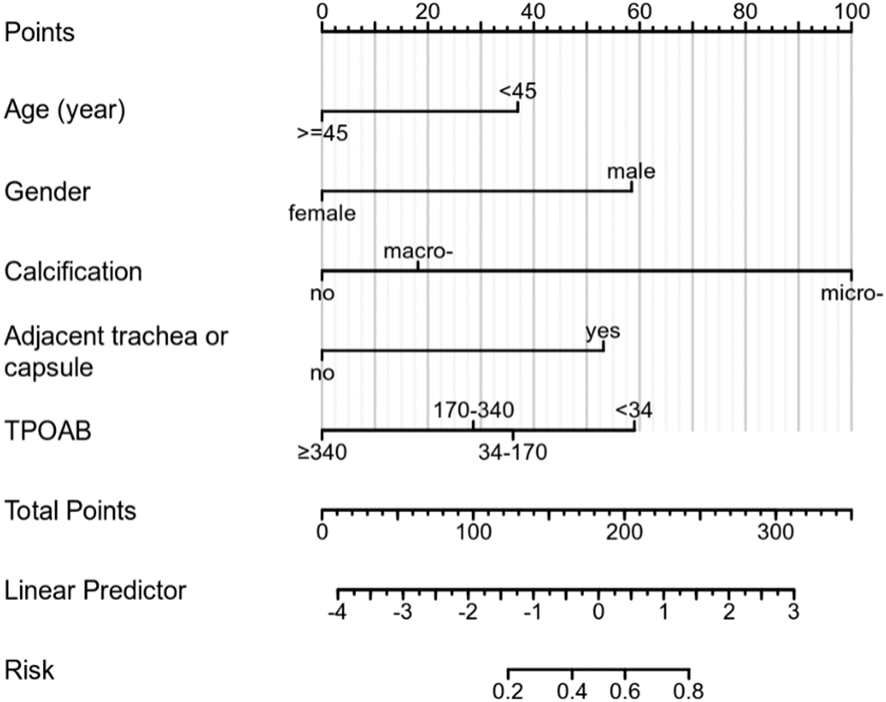
A nomogram for predicting CLNM in patients of PTMC with HT.

Based on multivariate regression analysis results, age, gender, calcification, adjacent to the trachea or capsule, and TPOAB level higher than 340 IU/mL were identified as independent predictors of CLNM in PTMC patients with HT. A nomogram was constructed using these variables to display corresponding CLNM risk characteristics and calculate ontogenetic CLNM risk values (**Figure 3**).

**Figure 4:**
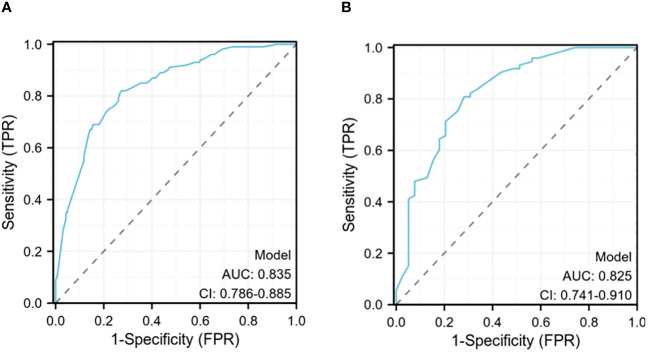
**(A)**The receiver operating characteristics (ROC) curve in the training cohort (AUC=0.835); **(B)** The receiver operating characteristics (ROC) curve in the validating cohort (AUC=0.825).

The ROC curve of the prediction model demonstrates that the AUC for the training cohort is 0.835 (95% CI: 0.786–0.885) (**Figure 4A**), while for the validation cohort, it is 0.825 (95% CI: 0.741–0.910) (**Figure 4B**). These results from both cohorts indicate a strong discriminatory ability of the model in predicting CLNM risk.

**Figure 5:**
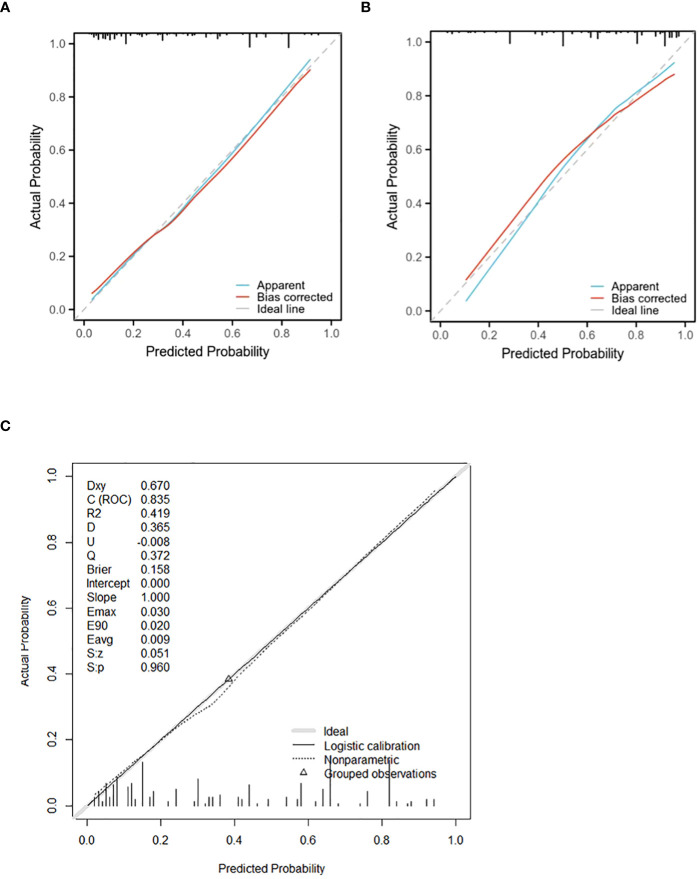
The calibration curve for evaluating the accuracy of the nomogram in the training cohort **(A)** and the validating cohort **(B)** (The mean absolute error is less than 0.05). **(C)**shows the calibration curve of the training cohort model (Brier score:0.158, slope:1.00). The calibration curve ranks all individuals from low to high probability predicted by the model, divided into 10 equal parts, with the mean of each equal predicted probability as the X-axis and the proportion of actual events occurring as the Y-axis. There are 3 lines in each graph, the blue line is the prediction curve of the model, the red line is the corrected curve, and the dashed line (45° slash) is the actual curve. A perfect prediction model’s curve should be moderate to the dashed line, meaning that the model’s prediction probability and the actual probability match perfectly.

The verification process was conducted on the validation cohort, yielding a *P*-value of 0.8719 for the Hosmer-Lemeshow goodness-of-fit test and a *P*-value less than 0.01 for the likelihood ratio test, confirming good calibration and differentiation abilities of our model. The C-index evaluation result was found to be 0.825, further supporting its accuracy. Through bootstrap analysis with resampling performed on both cohorts, calibration curves were generated (**Figure 5**). The average absolute error in predicted risk was less than 0.05 in both cohorts, demonstrating excellent consistency between corrected and observed metastasizing risks. This assessment using the Brier score yielded favorable values such as slope=1, E_90_ = 0.020, and E_50_ = 0.008.

**Figure 6:**
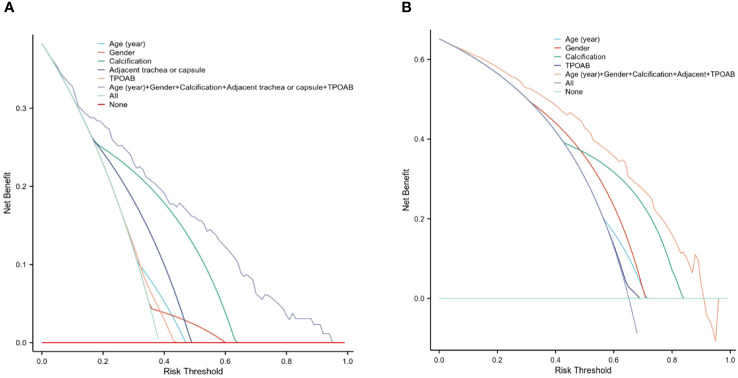
The DCA for evaluating the clinical application value of the model. The y-axis represents the net benefit different colors indicate different models. **(A)** When the threshold probability was 3.3% to 94.6% in the training cohort, the curve of the combined model was higher than that of other models, indicating a higher predictor of CLNM. **(B)** shows the DCA in the validation cohort.

In addition to this evaluation approach, we employed DCA reassessment modeling to assess the clinical practicability of CLNM prediction in PTMCs with HT. DCA revealed that when considering risk thresholds ranging from 3.3% to 94.6% for the training cohort (**Figure 6A**) and from4.5% to 90.9%for the validation cohort (**Figure 6B**), the overall net benefit provided by the nomogram model incorporating all factors surpassed that obtained through “no intervention” or “treat-all” strategies. Furthermore, the combined predictive power of all factors outperformed individual factor predictions, suggesting enhanced accuracy in assessing CLNM risk.

**Figure 7:**
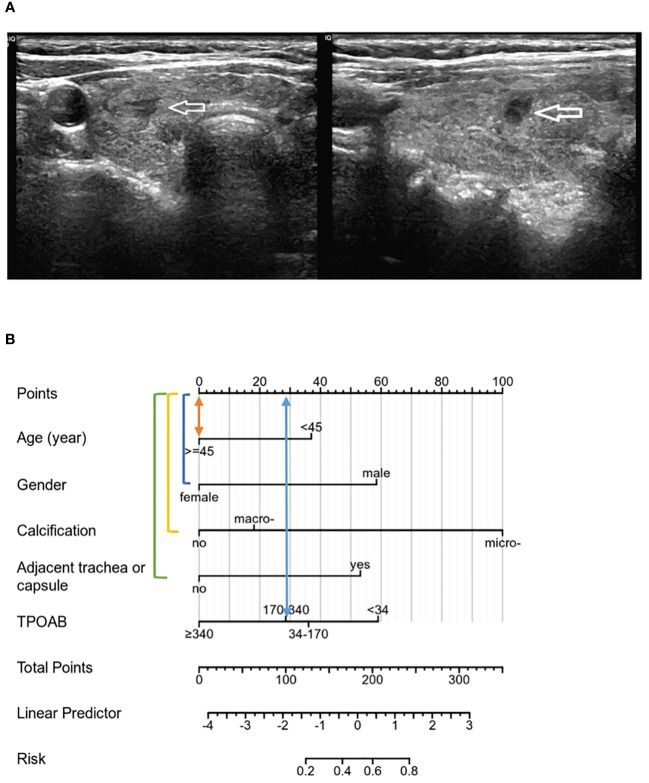
**(A)** PTMC lesions showed no significant calcification on ultrasound images, and were not adjacent to the trachea and capsule, and all risk factors were negative. TPOAB: 247.37 IU/mL. **(B)** According to the nomogram, the CLNM risk score was 28 points, indicating low risk (<20%), and no CLNM was confirmed by postoperative pathology.

**Figure 8:**
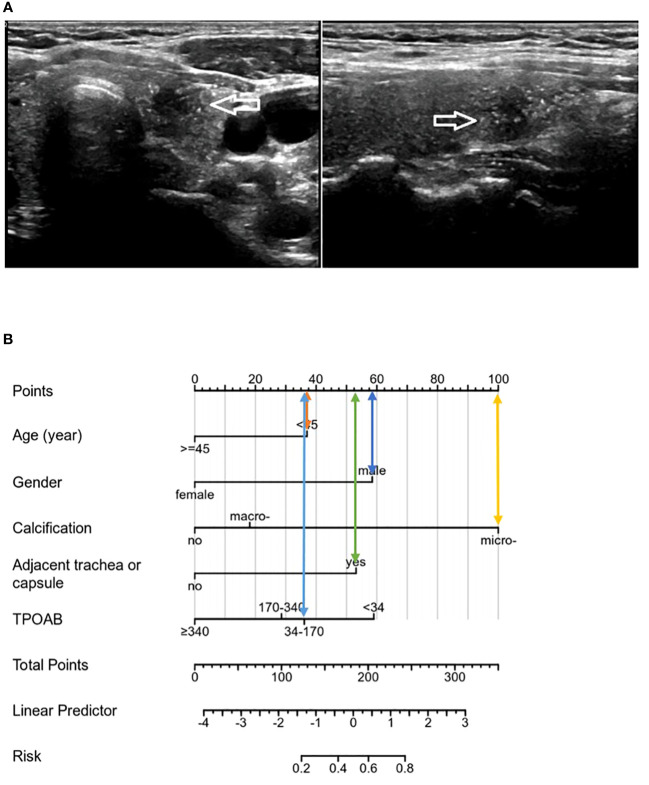
**(A)** The patient was a male with 35 years, and the tumor showed microcalcification on ultrasound, close to the trachea and capsule, TPOAB: 146.50 IU/mL. **(B)**. The risk score of the CLNM was 283 points, belonging to the high-risk group (>80%), and CLNM was finally confirmed by pathology.

The above validation demonstrated that the nomogram to evaluate CLNM risk in PTMCs with HT performed well, further confirming the robustness and accuracy of the model prediction. Two PTMC patients with HT in cN0 were selected to conduct individualized factor analysis and evaluate the risk of CLNM according to the prediction model of this study, as shown in **Figures 7**, **8**.

## Discussion

With the increasing prevalence of papillary thyroid microcarcinoma (PTMC) and excessive testing worldwide, accounting for over 50% of new cases of thyroid cancer (13), precise management of this disease has become crucial. The 2015 ATA guidelines recommend pCND as a management strategy for PTMC patients with clinically involved CLNs or stage T3 and T4 tumors. Immediate surgical intervention is advised for PTMCs with clinical lymph node metastasis, distant metastasis, invasion of neighboring organs, or other high-risk characteristics (14). In China (15), surgical intervention remains the primary treatment method for PTMC patients. Previous studies have reported that 38% of PTMC patients had CLNM (5). However, in our study, approximately 42% of patients exhibited CLNM. Among PTMCs with HT in cN0, latent CLNM was observed in 38.3%, while among those without HT, CLNM was present in 44%. Considering the potential impact on patient outcomes, pCND should be performed during initial thyroidectomy in high-risk individuals to avoid unnecessary re-surgery and associated complications commonly seen in recurrent cases. However, preoperative imaging has limited utility in detecting lymph node metastasis (16). Thus, accurate evaluation of actual CLNM status is necessary for cN0-stage patients.

Clinical observations indicate a frequent coexistence between HT and thyroid cancer (17), moreover, HT is associated with an increased prevalence of PTMC (18, 19). Studies have demonstrated that PTMCs with concomitant HT exhibit lower invasiveness and better prognosis (12) due to chronic immune inflammation which also affects CLNs’ characteristics. Additionally reported is the protective role played by HT against BRAF^V600E^ gene mutation occurrence as well as extrathyroidal extension and lymph node metastasis development. Thus, indicating that PTMCs with HT are at relatively low risk. Thyroidectomy and CLND are performed in patients who already have lymph node metastasis. By targeting the risk factors for latent central compartment lymph node metastasis (CLNM) in the subgroup of PTMCs with HT in cN0 and establishing a personalized model for this specific population, we can better determine which type of patient is more likely to experience CLNM.

Some predictive models evaluate CLNM in PTMCs but overlook the impact of HT on these individuals. In this study, we found that although the number of dissected PTMCs was higher among those with HT compared to those without it, the CLNMR was significantly lower. The relationship between HT and PTC remains controversial, however, current findings suggest that certain immune cells and factors associated with HT exert protective effects on thyroid cancer by destroying cancer cells through specific antigens while infiltrating the thyroid gland lesions and their respective lymphatic drainage channels (20–22). Our results align with this perspective by demonstrating that HT is not an independent risk factor for CLNM in PTMCs; rather when comparing patients without HT to those with it there was a significantly lower CLNMR (*P*<0.001), thereby confirming our previous hypothesis. The characteristics of PTMCs with and without HT differ, thus necessitating different approaches for analyzing CLNM status and implementing predictive CLNM management strategies. For instance, Lawrence A et al. (23) found that the risk of CLNM was significantly lower in females compared to males in studies on PTC/PTMC (2). This study also observed a higher propensity for CLNM in young males with HT; however, the proportion of males in the HT group was lower than that in the non-HT group (*P*<0.001). From a gender perspective, combined HT exhibited some protective effects against CLNM.

Screening PTMCs with HT in cN0 for relevant clinical features influencing CLNM yielded results consistent with those obtained from univariate analysis. Furthermore, multivariate analysis identified four independent risk factors (*P*<0.05) for CLNM: age under 45 years old, male gender, presence of calcification adjacent to trachea or capsule, and high levels of TPOAB as a protective factor. Previous studies have highlighted tumor size and bilateral lesions as important risk factors for CLNM in PTMCs (24–26); however, these variables did not retain statistical significance after multivariate analysis when considering PTMCs with HT specifically. Future discussions may consider grouping PTMCs based on tumor size. The role of TGAB and TPOAB in PTMC development and metastasis remains debated; nevertheless, it has been reported that elevated levels of TGAB and TPOAB are associated with HT which may contribute to thyroid cancer tumorigenesis and CLNM while also possessing prognostic value (27). High levels of TPOAB demonstrated a certain degree of protection against CLNs despite not showing significant effects during univariate analysis. Its inclusion into the multivariate analysis revealed a statistically significant association with reduced incidence of CLNM. Serum TPOAB levels were found to be higher among individuals with an underlying background of HT. These findings demonstrate the protective effect of HT on CLN, which is consistent with previous studies on CLNM (11, 28, 29). Huang et al. (30) identified microcalcification as a significant factor in CLNM among PTMCs.

Each gland lobe of the thyroid possesses its internal lymphatic system (31) and metastasis typically follows a pattern from the central ventricle to the ipsilateral cervical region before spreading to contralateral and distant lymph nodes. Our study revealed that adjacent to the trachea or capsule was associated with an increased risk of CLNM due to anatomical factors facilitating tumor cell infiltration and lymphatic drainage through deep anterior CLNs located in front and on both sides of the larynx surrounding the thyroid gland and trachea (32). Although tumor location (upper/middle/inferior) did not predict metastasis risk per se, it may determine the area affected by metastasis (33). Unlike previous studies that focused only on ultrasound features or perioperative laboratory results (34) for analysis, our study comprehensively considered clinical data when identifying independent risk factors (microcalcification, adjacent to trachea or capsule) predicting CLNM in PTMC patients with HT before surgery assessment.

Although TG has been partially shown to be related to the metastasis and prognosis of thyroid cancer, TGAB is elevated in most HT patients, and TGAB can affect TG levels, so this variable is not included at this stage. Unexpectedly, preoperative ultrasound assessment could determine independent risk factors (microcalcification, adjacent to the trachea or capsule) that ultimately predict CLNM in patients with PTMC and HT. Viola et al. (35) demonstrated that preoperative ultrasound assessment of CLNM (CN0/CN1) had limited sensitivity and no advantage. Some scholars have suggested that the proximity of CLNs to the trachea may interfere with ultrasound conduction due to air interference. This study addresses the limitation of accurately identifying lymph node status before surgery using ultrasound by leveraging ultrasonic characteristics for predicting CLNM risk. The model was evaluated through various validation results which consistently demonstrated good prediction consistency for metastasis risk compared to the nomogram. Additionally, the DCA from the training cohort effectively showcased its clinical practical value. This model can be flexibly applied in clinical diagnosis and treatment settings, providing more intuitive results. Based on these five identified factors, a precise and comprehensive assessment of CLNM risk was conducted for PTMCs with HT who were initially classified as cN0, enabling individualized treatment planning. Patients can be categorized into high-risk or low-risk groups based on their CLNM risk levels. For instance, PTMC patients at low risk for CLNM may benefit from active surveillance or minimally invasive ablation therapy as depicted in **Figure 7** above; whereas those at high risk due to multiple factors should consider thyroidectomy and pCND (**Figure 8**).

However, this study has certain limitations. Firstly, the retrospective nature of the analysis prevents accurate prediction of prospective analysis results. Secondly, the limited sample size and exclusive focus on Chinese population introduce a potential selection bias. To address this limitation, future studies should consider expanding the sample size. Thirdly, variations in surgical methods and regional anatomy among different surgeons may influence result interpretation. Nevertheless, compared to single-center non-comprehensive research studies, our study utilized samples from two centers to mitigate sample selection bias to some extent. We also considered comprehensive clinical research factors and quantitatively validated our model results, providing a relatively accurate basis for individual clinical decision-making in papillary thyroid carcinoma.

Given that nodular lymphatic systems flow widely in various directions for PTC patients (35), accurately predicting lymphatic drainage remains challenging. Therefore, further investigation into the rules governing lymph node metastasis is highly significant and holds promising prospects for development.

## Conclusion

In summary, this study revealed that PTMC patients with HT had lower involvement of CLNs compared to those without HT. CLNMR was decreased in these patients indicating a protective effect against CLNM and reduced tumor invasiveness. Thus, it is recommended that a specific prediction system be employed for PTMCs with HT to more accurately evaluate CLNM risk. Additionally, we discussed and confirmed that younger age (below 45 years), male, intralesional microcalcification, and adjacence to trachea or capsule were associated with increased likelihood of CLNM occurrence; conversely, higher titers of serum TPOAB exhibited a protective effect against CLNM. For stage cN0 PTMCs with HT, a predictive model can be established by combining ultrasound and clinical characteristics to determine the risk of latent CLNM before surgery and screen out high-risk patients who are more suitable for pCND, to accurately conduct individualized treatment management.

## Data availability statement

The raw data supporting the conclusions of this article will be made available by the authors, without undue reservation.

## Ethics statement

These studies involving humans were approved by the Ethics Committee of the First Affiliated Hospital of Shandong Province Medical University. The studies were conducted in accordance with the local legislation and institutional requirements. The ethics committee/institutional review board waived the requirement of written informed consent for participation from the participants or the participants’ legal guardians/next of kin because this study is a retrospective study. Written informed consent was not required from the individual(s) for the publication of any potentially identifiable images or data included in this article because this study does not contains identifiable human images or underage patients.

## Author contributions

YL: Conceptualization, Data curation, Formal analysis, Investigation, Methodology, Project administration, Software, Supervision, Writing – original draft, Writing – review & editing. NC: Data curation, Writing – review & editing. FL: Methodology, Supervision, Writing – review & editing. YW: Methodology, Supervision, Writing – review & editing. BW: Conceptualization, Data curation, Formal analysis, Investigation, Methodology, Software, Supervision, Validation, Writing – review & editing.
